# Understanding surgical care delivery in Sub-Saharan Africa: a cross-sectional analysis of surgical volume, operations, and financing at a tertiary referral hospital in rural Tanzania

**DOI:** 10.1186/s41256-019-0122-2

**Published:** 2019-10-26

**Authors:** Praveen Paul Rajaguru, Mubashir Alavi Jusabani, Honest Massawe, Rogers Temu, Neil Perry Sheth

**Affiliations:** 1 Center for Public Health Initiatives, University of Pennsylvania, Philadelphia, PA USA; 2 Department of Orthopaedic Surgery, Kilimanjaro Christian Medical Center, Moshi, Tanzania; 3 Department of Orthopaedic Surgery, University of Pennsylvania, Philadelphia, PA USA

**Keywords:** Global health systems, Global surgery delivery, Capacity building, Universal health coverage, Access to healthcare, Low- and middle-income countries, Financing healthcare, Systems and operations

## Abstract

**Background:**

Access to surgical care in Low- and Middle-Income Countries (LMICs) such as Tanzania is extremely limited. Northern Tanzania is served by a single tertiary referral hospital, Kilimanjaro Christian Medical Centre (KCMC). The surgical volumes, workflow, and payment mechanisms in this region have not been characterized. Understanding these factors is critical in expanding access to healthcare. The authors sought to evaluate the operations and financing of the main operating theaters at KCMC in Sub-Saharan Africa.

**Methods:**

The 2018 case volume and specialty distribution (general, orthopaedic, and gynecology) in the main operating theaters at KCMC was retrieved through retrospective review of operating report books. Detailed workflow (i.e. planned and cancelled cases, lengths of procedures, lengths of operating days) and financing data (patient payment methods) from the five KCMC operating theater logs were retrospectively reviewed for the available five-month period of March 2018 to July 2018. Descriptive statistics and statistical analysis were performed.

**Results:**

In 2018, the main operating theaters at KCMC performed 3817 total procedures, with elective procedures (2385) outnumbering emergency procedures (1432). General surgery (1927) was the most operated specialty, followed by orthopaedics (1371) and gynecology (519). In the five-month subset analysis period, just 54.6% of planned operating days were fully completed. There were 238 cancellations (20.8% of planned operations). Time constraints (31.1%, 74 cases) was the largest reason; lack of patient payment accounted for as many cancellations as unavailable equipment (6.3%, 15 cases each). Financing for elective theater cases included insurance 45.5% (418 patients), and cash 48.4% (445 patients).

**Conclusion:**

While surgical volume is high, there are non-physical inefficiencies in the system that can be addressed to reduce cancellations and improve capacity. Improving physical resources is not enough to improve access to care in this region, and likely in many LMIC settings. Patient financing and workflow will be critical considerations to truly improve access to surgical care.

## Background

Surgical care in many Low- and Middle-Income Countries (LMICs) is extremely limited, with millions lacking access; Tanzania is no exception [1]. A World Health Organization survey of the country’s primary medical centers found that of the 35 listed basic interventions, only suturing was routinely available at all facilities. These facilities represent 64 surgeons, across multiple sub-specialties, serving a catchment population of 23 million; they appear to lack the capability to deliver a full complement of surgical services [2]. Alternatively, referral hospitals in the country have a greater capacity to deliver emergency care, as they have a greater level of available infrastructure and equipment. Thus these tertiary referral facilities are more likely to provide surgical care in its entirety to all patients in need [3].

A focused approach on the northern corridor of Tanzania demonstrates a high surgical burden and need for access to care. This rural region consisting of the provinces of Kilimanjaro, Tanga, Arusha, Manyara, and Singida has a population of over 11 million people [4]. While there are aforementioned primary medical facilities and other smaller providers of care in the region, this enormous catchment area is served by just one major academic tertiary referral hospital, Kilimanjaro Christian Medical Centre (KCMC) [4]. Using orthopaedic surgery as an example, it has been estimated that more than 90% of the population lacks access to surgical care [5]. The Orthopaedic department at KCMC alone sees over 11,000 admitted and outpatient patients per year. Of the annual inpatient census, over 95% require surgical intervention of which less than 60% actually receive surgical care, with the average time to surgical intervention being greater than 10 days [6]. It is evident that a discrepancy exists between supply and demand; however, the specific reasons for this are currently not well understood.

While physical resources are limited, other factors such as issues regarding workflow at referral centers in Tanzania have previously been reported [7]. Patient financial burden has also been shown to play a major role in adequate care delivery in similar settings, such as Uganda [8]. Data from KCMC has previously quantified the orthopaedic burden of disease as well as the ability of patients in the surrounding region to access orthopaedic care, but little is known about the overall operating theater efficiency and utilization [4, 6]. In addition, operating theater case mix (i.e. orthopaedic, general, emergency, elective, etc.) and methods used by patients to pay for care are unclear, as they are not present in the academic literature. In our queries, we also found no Medline-indexed literature or any literature available through PubMed demonstrating an understanding of how operating theaters in LMICs function in terms of daily theater schedules and overall capacity.

This study retrospectively reviewed hospital data from KCMC to determine the main operating theater surgical burden, operations and workflow, and patient methods of payment. The purpose of this study was to evaluate the operations and financing of the main operating theaters at a tertiary referral hospital in Sub-Saharan Africa. We hypothesized that barriers limiting patient access to surgical care existed beyond the lack of physical resources in this setting.

## Methods

### Study setting and location

Data for this study was obtained from KCMC, a tertiary referral hospital in Sub-Saharan Africa. At KCMC, there are five main operating theaters: general surgery (Theater 1), multi-disciplinary emergency surgery (Theater 2), gynecology (Theater 3), orthopaedic surgery (Theater 4), and multi-disciplinary septic surgery (Theater 5). Operating theater records at KCMC are manually recorded at the conclusion of each case by the surgical staff and stored in administrative rooms in the surgical wards. Ethical clearance was obtained from the KCMC ethics committee prior to data collection and analysis.

### Measurements

Two surgical data sources were used for this study: overall operative volume logs and detailed operating theater logbooks.

#### Year-long data outcome measures

Retrospective operative volume logs were obtained, listing the volume and specialties of main operating theater procedures performed for the calendar year 2018. Data points were unavailable for 4 days (October 6–8, and November 4) which were excluded; all other data was included.

Operating report records were stratified by the three major specialties (general, gynecology and orthopaedics), not by operating theater. Thus, outcomes were based on specialty, the emergent or elective nature of the procedure and the day of week that the procedure was performed.

#### Five-month data outcome measures

We retrospectively collected data from operating theater logbooks for all surgical procedures performed in the KCMC main operating theaters during a five-month period between March 1, 2018 and July 31, 2018. Both data sets were collected and independently evaluated by two authors (PPR and MJ), and recorded in a de-identified, password protected Microsoft Excel spreadsheet for statistical analysis.

From this data set, we assessed the following: the number of planned versus completed surgical cases, anesthesia induction time (which defined the start of the day and each procedure), procedure end times, reasons for case cancellation, the day a procedure was performed (i.e. weekend or weekday), and the method of patient payment for elective procedures only. This information was used to determine the average number of procedures performed per operating day. For the emergency cases (theater #2), the number of cases performed, procedure length, and the day the procedure was performed was recorded.

### Statistical analysis

Descriptive and summary statistics were used to evaluate the outcome measures, including theater operating frequency and completion, operating day lengths, volumes of procedures by specialty, and payment methods observed.

Student’s t-tests were performed to analyze procedure length and operating day length differences between emergency and elective procedures and weekend (Saturday and Sunday) and weekday (Monday through Friday) procedures. Chi-square tests were performed to analyze differences between emergent and elective operating theaters, based on billing, volume, and cancellation reasons. Analysis of variance (ANOVA) tests were performed to analyze differences in procedure length by billing method and operating theater, and operating day length by operating theater.

For the five-month data analysis, given the retrospective nature of this review, data points were not always available. In these cases, we calculated the percentages of data point availability. For statistical analysis, missing data points were excluded.

For method of patient payment, if a patient could not pay for elective procedures or the method was not recorded by the staff, a payment method was not documented in the surgical log. For the purpose of payment method analysis, these cases were recorded as payment not listed (NL) (*n* = 51, 5.5%) and were grouped with the explicitly listed charity care cases (*n* = 5, 0.5%) for a combined NL/Other sample (*n* = 56, 6.1%).

Statistical analysis was performed using Stata IC Release 15 (StataCorp LLC, College Station, Texas). All tests were two-sided, and the statistically significant two-tailed *p*-value was set at 0.05 a priori.

## Results

### Case volume and distribution

In 2018, the five main operating theaters at KCMC performed 3817 procedures, with elective procedures (2385, 62%) outnumbering emergency surgical procedures (1432, 38%). General surgery performed the most procedures (1927), but orthopaedics had the greatest percentage of procedures performed on weekends (37.6%) (Table 1).

**Table 1:** Total recorded case volume in 2018 (361 days)

	# Emergency	# Elective	# Total
# General Surgery	809	1118	1927
# Weekday	582 (71.9%)	956 (85.5%)	1538 (79.8%)
# Weekend	227 (28.1%)	162 (14.5%)	389 (20.2%)
#Orthopaedics	464	907	1371
# Weekday	348 (75.0%)	507 (55.9%)	855 (62.4%)
# Weekend	116 (25.0%)	400 (44.1%)	516 (37.6%)
# Gynecology	159	360	519
# Weekday	106 (66.7%)	263 (73.1%)	369 (71.1%)
# Weekend	53 (33.3%)	97 (26.9%)	150 (28.9%)
#Total	1432	2385	3817
# Weekday	1036 (72.3%)	1726 (72.4%)	2762 (72.4%)
# Weekend	396 (27.7%)	659 (27.6%)	1055 (27.6%)

During the 5-month period of March 1, 2018 to July 31, 2018, 589 (39%) cases were completed in the emergency operating theater and 919 (61%) cases were performed in the elective theaters, for a total of 1508 procedures (Table 2). The orthopaedic theater had the largest surgical volume of all of the elective theaters (323), while the emergency theater exhibited the largest overall case volume. There was statistically significant variation among the balance of weekday and weekend cases by operating theaters (*p* < 0.001). The busier orthopaedic and emergency theaters completed nearly one third of their cases on weekends, while less than one eighth of cases were performed on weekends in the other theaters.

**Table 2:** Five-month procedure volume analysis

	# Cases weekday	#Cases weekend	*P*-Value^•^	Total
# Cases Theater 1 (General)	241 (98.8%)	3 (1.2%)	< 0.0001^*^	244
# Cases Theater 2 (Emergency)	416 (70.6%)	173 (29.4%)		589
# Cases Theater 3 (Gynecology)	144 (100%)	0 (0.0%)		144
# Cases Theater 4 (Orthopaedics)	219 (67.8%)	104 (32.2%)		323
# Cases Theater 5 (Septic)	185 (88.9%)	23 (11.1%)		208
Total	1205 (79.9%)	303 (20.1%)		1508

^•^χ2 Theater by Day Analysis

*Statistically significant, *p*<0.05

### Efficiency and procedure completion

Emergency operations (98.0% of calendar days) were performed on more days than elective procedures (67.0% of calendar days), despite the finding that the majority of cases were elective (Table 3). Regarding elective procedures, the orthopaedic theater had the highest operating rate (79.7% of calendar days), or 65% more than the gynecology theater (48.4% of calendar days). Yet despite the lower operative rate, the gynecology theater had the highest rate of planned schedule completion (64.9% of operating days), while orthopaedics was near the bottom in schedule completion (51.6% of operating days) (Table 3).

**Table 3:** Eligible operating days with completion

	Calendar days	Operating days	Percent of eligible days operated	Fully completed operating days	Percent of fully completed operating days	*P*-Value^•^
Total						
Emergency						
Emergency	153	150	98.0%	N/A	N/A	N/A
Elective	612	410	67.0%	224	54.6%	
Theater						
1 (General)	153	104	68.0%	50	48.1%	N/A
2 (Emergency)	153	150	98.0%	N/A	N/A	
3 (Gynecology)	153	74	48.4%	48	64.9%	
4 (Orthopaedics)	153	122	79.7%	63	51.6%	
5 (Septic)	153	110	71.9%	63	57.3%	
Weekend						
Yes	220	81	36.8%	N/A	N/A	N/A
No	545	479	87.9%	N/A	N/A	
Elective						
Theater						
1 (General)	153	104	68.0%	50	48.1%	0.128
3 (Gynecology)	153	74	48.4%	48	64.9%	
4 (Orthopaedics)	153	122	79.7%	63	51.6%	
5 (Septic)	153	110	71.9%	63	57.3%	
Weekend						
Yes	176	37	21.0%	27	73.0%	0.019*
No	436	373	85.6%	197	52.8%	
Total	765	560	73.2%	N/A	N/A	N/A

^•^χ2 analysis Fully Completed Days by Emergency, Theater, Weekend status; tests n/a when fully completed operating days are not measurable (i.e. when emergency cases are in the dataset)

*Statistically significant, *p*<0.05

Despite less frequent scheduling, efficiency was higher on weekends as measured by cancellations, total case volume and completion of all planned cases on a given operative day. Planned weekends, while operating on only 21.0% of eligible calendar days, were fully completed on 73.0% of operative days; for comparison, weekdays were operated on 85.6% of calendar days, but were fully completed just 52.6% of the time (Table 3). Cases were scheduled for a total of 410 operating days across the four elective theaters, and 8 days were entirely cancelled; all were weekdays. More procedures were planned and completed at a higher rate on weekend operating days compared to weekday operating days (Table 4). Orthopaedics also had the highest number of planned procedures and completed procedures per operating day, despite low completion of procedures (Table 4). This indicated a large elective orthopaedic burden on the main operating theaters.

**Table 4:** Elective procedure volume planned vs. completion per operating day

	Planned procedures mean	Planned procedures STDEV	*P*-value^•^	Completed procedures mean	Completed procedures STDEV	*P*-value^••^	Average completion percentage
Theater							
1 (General)	2.89	1.03	< 0.0001*	2.35	1.06	< 0.0001*	81.3%
				1.95	0.76		81.9%
3 (Gynecology)	2.38	0.92		2.65	1.50		81.5%
4 (Orthopaedics)	3.25	1.42		1.89	0.79		76.8%
5 (Septic)	2.46	0.96					
Weekend							
Yes	3.76	2.47	< 0.0001*	3.51	2.24	< 0.0001*	93.4%
No	2.70	0.92		2.12	0.89		78.5%
All Complete							
Yes	2.47	1.14	< 0.0001*	2.47	1.14	< 0.0001*	100.0%
No	3.18	1.10		1.97	1.10		61.9%
Total	2.79	1.18		2.24	1.15		80.3%

^•^ANOVA or T-Test analysis of # Planned procedures by Emergency, Theater, Weekend status

^••^ANOVA or T-Test analysis of # Completed procedures by Emergency, Theater, Weekend status

*Statistically significant, *p*<0.05

### Daily workflow and cancellations

On a given day, the average elective operating theater day was just 5 h and 25 min long from 9:44 AM to 3:09 PM (Table 5), with an average planned volume of 2.79 procedures per day. Weekend operating days were still significantly shorter than weekday operating days (4 h, 34 min vs 5 h, 29 min). The orthopaedic theater operating days were the longest of the elective theaters at 6 h and 12 min, while none of the others were beyond 6 h.

**Table 5:** Elective operating day lengths

	Operating day length mean (Hours: Minutes)	Operating day length STDEV (Hours: Minutes)	*P*-value^•^	Availability of data points^••^
Theater				
1 (General)	5:52	2:13	< 0.0001*	97.1% (101/104)
3 (Gynecology)	5:02	2:26		95.9% (71/74)
4 (Orthopaedics)	6:12	2:02		
5 (Septic)	4:20	2:13		95.1% (116/122)
				94.5% (104/110)
Weekend				
Yes	4:34	2:33	0.0282*	91.9% (34/37)
No	5:29	2:17		96.0% (358/373)
All Complete				
Yes	5:42	2:23	0.0069*	97.3% (218/224)
No	5:03	2:12		93.5% (174/186)
Total	5:25	2:19		95.6% (392/410)

^•^ANOVA or T-Test analysis of Operating Day Length by Theater, Weekend status, and whether all procedures were complete on a given day

^••^The numerator is the number of operating days for which this time data was available; the denominator is the number of operating days total for the given condition

*Statistically significant, *p*<0.05

The reasons for case cancellation are depicted in Table 6. One in five planned elective cases were cancelled, although reasons for these cancellations were relatively consistent across the sub-specialties (*p* = 0.171). Time constraints (31.1%, 74 cases) and inadequate preparation (8.0%, 19 cases) were the largest non-medical causes. Patient inability to pay accounted for the same percentage of cancellations as a lack of equipment (6.3%, 15 cases each).

**Table 6:** Number of cancellations by elective theater

	1 (General)	3 (Gynecology)	4 (Orthopaedics)	5 (Septic)	Total	*P*-Value^•^
Time Constraints	23 (35.9%)	12 (36.4%)	25 (32.9%)	14 (21.5%)	74 (31.1%)	0.171
Medical	14 (21.9%)	7 (21.2%)	20 (26.3%)	11 (16.9%)	52 (21.8%)	
Inadequate Preparation	5 (7.8%)	4 (12.1%)	4 (5.3%)	6 (9.2%)	19 (8.0%)	
Patient Not Paid	4 (6.3%)	0 (0.0%)	3 (3.9%)	8 (12.3%)	15 (6.3%)	
Equipment Unavailable	4 (6.3%)	3 (9.1%)	7 (9.2%)	1 (1.5%)	15 (6.3%)	
Patient Noncompliant	1 (1.6%)	1 (3.0%)	5 (6.6%)	5 (7.7%)	12 (5.0%)	
Case Done Elsewhere	3 (4.7%)	2 (6.1%)	1 (1.3%)	4 (6.2%)	10 (4.2%)	
Emergency	1 (1.6%)	3 (9.1%)	0 (0.0%)	5 (7.7%)	9 (3.8%)	
Transfusion Lacking	3 (4.7%)	0 (0.0%)	1 (1.3%)	2 (3.1%)	6 (2.5%)	
Other	1 (1.6%)	1 (3.0%)	2 (2.6%)	3 (4.6%)	7 (2.9%)	
Reason Not Given	5 (7.8%)	0 (0.0%)	8 (10.5%)	6 (9.2%)	19 (8.0%)	
Total	64 (100%)	33 (100%)	76 (100%)	65 (100%)	238 (100%)	

^•^χ2 analysis of Cancellation Reason by Theater

### Patient payment

Sources of financing for elective theater cases (Table 7) included insurance (45.5%, 418 patients) and cash (48.4%, 445 patients). Fifty-six (6.1%) patients had no financing listed or were financed by welfare. Operating theaters exhibited different payment schemes (*p* = 0.021), as the orthopaedic and septic theater cases were paid for in cash in a majority of cases, while the general surgery theater cases were more commonly financed through insurance.

**Table 7:** Elective room number of cases using various payment methods

	Cash	Insurance	NL/Other	Total	*P*-Value^•^
Theater					
1 (General)	109 (44.7%)	124 (50.1%)	11 (4.5%)	244	0.021*
3 (Gynecology)	70 (48.6%)	71 (49.3%)	3 (2.1%)	144	
4 (Orthopaedics)	161 (49.8%)	132 (40.9%)	30 (9.3%)	323	
5 (Septic)	105 (50.5%)	91 (43.8%)	12 (5.8%)	208	
Weekend					
Yes	56 (43.1%)	60 (46.2%)	14 (10.8%)	130	0.042*
No	389 (49.3%)	358 (45.4%)	42 (5.3%)	789	
Total	445 (48.4%)	418 (45.5%)	56 (6.1%)	919	

^•^χ2 analysis of Payment Mechanism by Theater and Weekend status

*Statistically significant, *p*<0.05

Variation was observed regarding weekend and weekday procedures (*p* = 0.042). Weekend procedures were mostly insured or NL/Other, compared to weekday procedures (57.0% vs 40.7%). Five patients (0.5%) were specifically listed as funded by KCMC social welfare; all were weekday cases from theaters 1, 2, and 5 (data not shown). Variation was observed in procedure length based on payment method differences (*p* = 0.010) with insured procedures (1 h, 58 min) being the shortest; Cash (2 h, 7 min) and NL/Other (2 h, 22 min) procedures were longer (Additional file 1: Table S1).

### Five month data availability

For the five-month data analysis, given the retrospective nature of this review, data points were not always available. In these cases, we calculated the percentages of data point availability. The lowest observed data point availability was 85.2% (for emergency theater procedure lengths), and most ranged above 90%, implying high availability of data points.

## Discussion

We evaluated the case distribution, workflow and operations, and financing of the main operating theaters at a tertiary referral hospital in Tanzania. Our results demonstrated that there are significant barriers to accessing care in this region, which include orthopaedic theater overflow, a high cancellation rate, inefficient workflow, and the inability for patients to pay for services. These issues cannot be fixed by an influx of physical resources alone, but will require changes at the system level in order to optimize patient access, procedure throughput, care delivery, and overall operations. Optimization of operations and scheduling could functionally improve access to care by allowing for optimal usage of physical resources. Further, systemic changes to reduce patient financial burden could decrease case cancellations substantially. We further describe the reasons for this as well as potential routes forward below.

The distribution of cases in the main operating theaters at KCMC demonstrated a high burden of general and orthopaedic cases, together accounting for 86.3% (3298) of cases in 2018. While general surgery was the most operated specialty, our analysis of a five-month period in 2018 found that the orthopaedic operating theater had the highest burden of elective procedures, had the highest number of total elective operating days, exhibited the most planned and completed procedures per operating day, and had the largest overall volume of elective procedures. Despite this, as well as averaging more procedures over the weekend than on weekdays and having the longest operative day, the orthopaedic theater was at the bottom in completing all planned procedures in a given day.

Despite being the busiest theater, the orthopaedic theater was also among the least efficient elective theaters. This may have been attributable to two problems: a large orthopaedic burden and a lack of capacity (i.e. time constraints) to perform all of these procedures during the week. Additional dedicated orthopaedic trauma operating rooms in the US have created enormous cost reductions and increased capacity to address the orthopaedic trauma burden [9]. As such, increasing the number of orthopaedic operating rooms may reduce operating theater burden and improve overall surgical capacity at KCMC. An Orthopaedic Center of Excellence in this region would similarly help to address the significant surgical burden currently presenting to KCMC [10].

Decreasing cancellation rates at KCMC may also optimize patient care. The observed cancellation rate was 20.9%. For comparison, the public United States Veterans Affairs (VA) system cancellation rate has been estimated at 12.4% [11]. The largest contributing factor at KCMC was not enough time on a given operative day (31.1%). This differed from studies at the public US VA medical system, where the largest reason for cancellation was on the demand side, or patient factors such as no-shows and cancellations due to self-rescheduling [11]. However, relative to other hospitals in Sub-Saharan Africa, KCMC appears to perform well. For example, a major tertiary hospital in Malawi was shown to have a cancellation rate of 44.2%, with major reasons including equipment shortages (50.9%) and time constraints (33.3%) [12]. Another Tanzanian hospital, Bugando Medical Centre, was found to have a cancellation rate of 21.0%, with major reasons including lack of theater space (53.0%) and equipment (28.4%) [13]. As such, KCMC appears to perform well compared to other African hospitals, and has a much lower rate of cancellation due to lack of equipment (6.3%). This increases the importance of improving workflows specifically in KCMC to reduce cancellations, as it appears to have an advantage in infrastructure compared to other hospitals studied in Sub-Saharan Africa.

Closer analysis of operating days supported the need for improved workflows; just half of planned elective operating days were completed at KCMC. This becomes an even greater issue when taking into account the academic role that KCMC plays in training the next generation of surgeons, which may result in lengthening of some procedures [5, 14]. Ensuring high case volume is critical to improving patient outcomes, decreasing complication rates and developing an adequate level of trainee proficiency [15–17].

When coupled with limited weekend operations, late start times, and early end times, a potential hypothesis for time constraint related cancellations could be due to workflow inefficiencies. This was supported by the finding that operating day start time was significantly earlier on days when all procedures were completed, compared to days when all procedures were not completed (9:38 AM vs 9:52 AM, *p* = 0.008). Furthermore, ending time was significantly later on days when all procedures were completed (3:21 PM vs 2:54 PM, *p* = 0.046). Potential improvements may involve flexible scheduling accounting for the demands of the various surgical departments together, not independently, through a Master Scheduling System [18, 19]. Accounting for case complexity by avoiding multiple long complex cases from being scheduled on a single day and adhering to planned start times can also assist in case completion in a cost-effective manner [20, 21].

Other opportunities may include improving communication and workflow between the surgical departments and the intensive care unit or promoting utilization of open operating theaters of lower-volume specialties (such gynecology) to account for overflow in higher volume specialties [19, 22]. This is especially relevant when understanding the surgical landscape at KCMC. All three specialties - gynecology, orthopaedics, and general surgery - had their own theaters. This equitable distribution was made despite disparities in volume, with gynecology operating on fewer than half of the eligible days and with a much lower volume than the other specialties. While some high-volume specialties had cases done in the emergency or septic theaters, the performance of the orthopaedic theater in particular demonstrates that this accommodation was not enough. Shifting some volume to available spaces, rather than scheduling strictly by specialty, may reduce time constraint related cancellations [21].

Our findings also implicated financing of care as a barrier to accessing care in North Tanzania. Analysis of a government facility in Uganda discovered that less than 5% of patients could access necessary surgical care without incurring catastrophic out-of-pocket expenditure. Accessing surgical care in LMICs such as Uganda or Tanzania can represent a substantial economic burden to a majority of the population [8]. While physical capacity and equipment limitations have been commonly discussed as barriers to care in low resource settings, at KCMC the number of surgical procedures cancelled due to unavailable equipment was equivalent to cancellations due to a patient’s inability to pay.

Insurance enrollment seemed to open the door to surgical care in Northern Tanzania. More than 45% of patients receiving elective surgical services at KCMC were insured. Compared to an estimate of 16% insurance coverage in the general population in Tanzania, those voluntarily accessing surgical care in this region were disproportionately insured [23]. This is consistent with other studies finding higher insurance utilization by higher socioeconomic status populations in Tanzania and may speak to a need to improve access to care for the disadvantaged [24]. In using procedure length as a proxy for the complexity of cases, insurance was used for significantly shorter and thus, on average, less severe cases. Insurance coverage was thus critical in receiving less complex care that may still have a large impact on quality of life. Receiving weekend care to prevent missing work and losing additional income required insurance coverage to an even higher degree. Such a disparity suggests that people receiving care are disproportionately better-off than the general population and will sacrifice fewer working days to access surgical care.

Yet despite the high level of insurance coverage observed, the most common payment method was cash. With a GDP per capita of 2,275,601 Tanzanian Shillings (TZS) ($983, conversion rate of $1 USD = 2315 TZS as of 4/15/2019), paying for care out of pocket at KCMC could be rather expensive for the average Tanzanian worker assuming the loss of income for hospitalization and required healthcare payments [25]. At KCMC, the cost of receiving an operation was priced at 250,000 TZS ($108), or more than 1 month of labor for the average Tanzanian; this did not include costs of imaging, implants and medication, or accommodations.

The World Health Organization has advocated for Universal Health Coverage (UHC) in every nation by 2030 [26]. This push has reached Tanzania as well; the Tanzanian government is working on legislation to improve national insurance coverage [27]. Based on our findings in the rural northern corridor of the country, this approach seems critical to improving access to care for all Tanzanians.

This study had several limitations. Its retrospective nature made us reliant upon previously collected written data since KCMC does not utilize electronic health records. However, the majority of data points were available and points of data collection were reviewed to ensure accuracy of data retrieval from these official hospital records. For method of patient payment, if a patient could not pay for elective procedures or the method was not recorded by the staff, a payment method was not documented in the surgical log. It was impossible to retrospectively determine whether all of these omissions or a specific ratio of the omissions were due to lack of patient payment or lack of recording. However, these accounted for a very small portion of overall financing (6.1% overall, Table 7). Future studies, particularly if carried out prospectively, should better ensure the data collection process. Another potential limitation is the sharing of rooms by specialties. However, hospital policy is to schedule cases by specialty, and we found just ten cases of 1508 that were listed as done in another room. As such, our results appear to be reflective of delivery of care by specialty, mitigating effects of misclassification bias.

The study also only looked at payment characteristics of patients who underwent surgical procedures; billing amounts were unavailable. As such, we only studied how patients paid for surgical care, not the amount paid. This gap will need to be addressed in future research to determine the degree of economic burden placed on patients seeking surgical care in this setting. The study was only performed at a major tertiary referral hospital in Tanzania, and the specific findings may not be generalizable to other LMICs. However, the findings did raise the importance of analyzing important features such as operative volume, workflow and operations, and patient payment in other LMIC settings.

This study should provide a blueprint for similar work to be done in other geographic regions, to accurately depict surgical care delivery and identify region-specific limitations and needs. Future studies should expand upon this work. While this study demonstrated the payment methods utilized for surgical care in Northern Tanzania, future studies should examine the socioeconomic burden placed on surgical patients for the same region. It is also unclear how workflows and care delivery are characterized for other medical services, such as inpatient or outpatient medicine, physiotherapy, radiology, and others. Understanding these aspects can define system level changes that must be implemented to improve access to holistic medical care in similar regions and other LMICs.

## Conclusion

Kilimanjaro Christian Medical Centre is a key hub for surgical care and education in the Sub-Saharan nation of Tanzania. This study demonstrated characteristics of the surgical volume, patient payment methods, and workflow aspects of surgical delivery in Northern Tanzania. We observed inefficiencies that can be addressed to reduce case cancellations and improve capacity for the benefit of patients accessing surgical care. Improving resources is not enough to improve access to care - understanding the distribution of volume, workflow and operations, and patient financing are critical considerations to truly improve access to surgical care.

## Supplementary information

**Additional file 1: Table S1.** Procedure Length Analysis.

## Data Availability

The dataset used and analyzed during the current study is available from the corresponding author on reasonable request.

## Abbreviations

KCMC: Kilimanjaro Christian Medical Centre
LMICs: Low- and middle-income countries
TZS: Tanzanian Shillings
UHC: Universal health coverage

## Supplementary information

**Supplementary information**accompanies this paper at 10.1186/s41256-019-0122-2.
