# Supplementary material for: Understanding surgical care delivery in Sub-Saharan Africa: a cross-sectional analysis of surgical volume, operations, and financing at a tertiary referral hospital in rural Tanzania
Source: Glob Health Res Policy. 2019 Oct 26;4:30. doi: 10.1186/s41256-019-0122-2 (PMC6816166; doi:10.1186/s41256-019-0122-2)
Supplement: Supplementary file 1 — Additional file 1: Table S1. Procedure Length Analysis. [file 41256_2019_122_MOESM1_ESM.docx]

**Additional Files**

- Table S1. Procedure Length Analysis
- Procedure Length Analysis
- Detailed analysis of the lengths of procedures based on various factors; referenced in manuscript

|  | **Procedure Length Mean (Hours: Minutes)** | **Procedure Length STDEV**  **(Hours: Minutes)** | **P-value^•^** | **Availability of Length Data Points^••^** |
| --- | --- | --- | --- | --- |
| **All Procedures Analysis** |  |  |  |  |
| **Emergency**  Emergency  Elective | 2:14  2:04 | 1:17  1:04 | 0.0079* | 85.2% (502/589)  93.9% (863/919) |
| **All Theaters**  1  2  3  4  5 | 2:08  2:14  2:10  2:09  1:49 | 1:20  1:17  0:57  0:59  0:54 | 0.0004* | 93.0% (227/244)  85.2% (502/589)  96.5% (139/144)  91.0% (294/323)  97.6% (203/208) |
| **Day**  Weekday  Weekend | 2:10  1:58 | 1:10  1:07 | 0.0129* | 91.2% (1099/1,205)  87.8% (266/303) |
| **Elective Analysis** |  |  |  |  |
| **Payment**  Insurance  Cash  NL/Other | 1:58  2:07  2:22 | 1:06  1:00  1:15 | 0.0101* | 92.3% (386/418)  95.5% (425/445)  92.9% (52/56) |
| **Theater**  1  3  4  5 | 2:08  2:10  2:09  1:49 | 1:20  0:57  0:59  0:54 | 0.0014* | 93.0% (227/244)  96.5% (139/144)  91.0% (294/323)  97.6% (203/208) |
| **Day**  Weekday  Weekend | 2:08  1:38 | 1:05  0:51 | <0.0001* | 94.8% (748/789)  88.5% (115/130) |
| **Total** | 2:04 | 1:04 |  | 93.9% (863/919) |
| **^•^**ANOVA or T-Test analysis of Procedure Length by Emergency, Theater, Weekend, and Payment status  **^••^** The numerator is the number of procedures for which this time data was available; the denominator is the number of procedures total for the given condition | | | | |
